# Euthanasia in Phthisis

**Published:** 1882-03

**Authors:** E. W. Berridge

**Affiliations:** London


### EUTHANASIA IN PHTHISIS.

E. W. Berridge, M. D., London.

In the 1881 session of the International Hahnemannian Association, the following resolution was presented by myself, and adopted by the Association:

"*Resolved*, that in incurable cases, we believe that the utmost possible relief from suffering is obtained from the *Simillimum*, and that antipathic or allopathic palliatives are not only unnecessary but injurious."

It is a common plea of the mongrels, to justify their departures from our law, that the *simillimum* only avails when a cure is possible, and that when the patient is necessarily incurable, nothing but allopathic palliatives can be relied on. This is strange logic, most worthy of the advocates of the new system of "Through the Looking-Glass Pathology" (see *The Organon*, vol. 1, pp. 321–2). Rational physicians would be likely to conclude that to *cure* was a greater work than to *relieve*, and that if Homœopathy could effect the greater, it could surely also effect the less. But as these pseudo-pathologists, who are ignorant of the natural course of an abscess (see *The Organon*, vol. 1, pp. 321–2), may be reasonably supposed to be ignorant of other important matters also, it may be well to call their attention to the following rule of practice:

*In cases where the vital powers are sufficiently vigorous to render a recovery possible under homœopathic treatment, the curative action of the remedy, once fairly established, continues for some time; but where they have sunk so low that death is inevitable, the action of the best-selected remedies, though the relief given by them is truly wonderful, is speedily exhausted, so that new remedies have to be continually selected, according to the continually changing symptoms.*

If, therefore, those who are ignorant equally of the nature of disease and of the action of medicines, neglect this rule, and in incurable cases give a course of medicines to be continued for several days, they will be sure to meet with failure; whereas, a careful examination of the patient's symptoms, at short intervals, combined with a careful selection of the remedy strictly according to the law of *similia similibus curantur*, will achieve the greatest possible amount of success. Let those who doubt the greater efficacy of Homœopathy in such cases, listen to the recital of the sufferings of those who are dying a daily death under the combined influences of the disease and narcotics; no relief, even for a time, being possible, but from drugs, whose reaction finally leaves the patient in a worse state than before. And let any who are tempted to use the nosodes *unscientifically, i.e.* on any principle but that of our infallible law, notice that I commenced the treatment with *syphilinum*, though there were no indications of syphilis, because the symptoms of that particular patient at that particular time indicated it; and though the disease was tuberculosis, I did not use *tuberculinum*, because it was not indicated by the symptoms of the patient at any time, while the other medicines were clearly indicated.

Miss D., æt. 30. Had been suffering from phthisis for six years. She was at first under allopathic, and then under pseudo-homœopathic treatment. I first saw her Oct. 19th, 1881. She was then taking two medicines in alternation, both having a strong odor, and one a bitter taste. They had only made her feel worse. I found her in the last stage of phthisis. In the right chest there was sibilus on inspiration, and bronchial breathing on expiration; on the left chest there were the same sounds, with the addition of great dullness or percussion in the upper part. Cough and dyspnœa come on after her midday dinner, has to fight for her breath; feels as if she would be suffocated. These symptoms last all night, and *at daybreak they get better* and she falls asleep. Sputa, yellow, thick, seem to come from left upper chest; must keep her hand pressed there when she coughs, as it enables to raise the phlegm more readily Has not left her bed for two days.

The italicized symptom being the great keynote for *syphilinum*, I gave the DM (F. C.) every four hours.

Oct. 21st. Marked improvement after first dose; had a better night; less cough; suffocation nearly gone; yesterday could sit up and felt stronger, to-day feels weaker and faint; last night's sleep not quite so good; cough and breathing a little more troublesome than yesterday. As the speedy improvement had been followed by a partial relapse, and yet no symptom indicating a new medicine had arisen, I gave no medicine.

Oct. 22d. Cough did not trouble her till 4 A. M. Suffocative attacks returned yesterday afternoon. She still gets better at daybreak, but the relief is not so marked as before. Sinking at stomach, causing faintness. Tongue dry; during faintness, mouth and throat feel parched. Since yesterday, loss of breath *on going to*

*sleep*, "as if the breath was taken all out of her." Suffocative feeling if the room is hot; wants to be fanned. Flushes of heat from head to chest. Thick yellow sputa. On coughing, pain like a knife in left upper chest, relieved by pressure of hand there. The former keynote had now nearly disappeared, and new symptoms of importance had arisen. Dyspnœa on falling asleep is given by Bœnninghausen under (*Bry.*) *Nux mosch.* and *Ran. bulb.*

Hering's *Guiding Symptoms* gives it under *Amm. carb.* (section 37), *Badiaga* (section 26), and *Carb. veg.* (section 26); this last being erroneously given in Jahr's *German Repertory*, vol. 1, p. 152, as *Carb. an.* It is also found in the proving of *Grindelia Robusta.* Hale states that it has been relieved by *Arsen.*, *Ignat.*, *Laches.*, *Nux Strychnia*, *Eucalyptus*, and *Ant. tart.*, but gives no authorities for his statement. I have verified it in my own practice under *Amm. carb*, and *Bryon.*, and Dr. David Wilson has verified it under the latter remedy also. Of these remedies, only *Amm. carb.*, *Arsen.*, and *Bryon.*, have aggravation of breathing from heat; and of these only *Bryon.* has relief to the pain of the cough and pressure. I gave *Bryon.* 103 M (F. C.) every four hours.

Oct. 23d., 7.15 P. M. Says that the first two or three doses did her much good, and she slept in afternoon and in night. The dyspnœa on falling asleep has ceased; but she has suffocative feeling now, even when awake. Cough was troublesome last night, it is now *relieved by lying on left side.* The sinking in stomach and faintness were better, but returned to-day at noon. Tongue dry. The flushes of heat have been better, but returned to-day at noon, worse than ever, from head to chest. She feels generally worse about noon. Breathing worse from lying on the back. The cutting in left chest has been much better, and *has ceased* since 5 P. M., *when she began to lie on left side.* Craving for sweet ale all day. Took last dose of *Bryon.* at 3.30 P. M. In Lippe's excellent *Repertory*, I found "cough worse lying on right side" (which is equivalent to "amelioration when lying on left side"), under *Amm. mur.*, *Carb. an.*, *Cina.*, *Stannum;* also "pain in chest worse when lying on sound side" (equivalent to "relief by lying on painful side"), under *Stannum.* The craving for ale I regarded as a demand of nature which should be gratified rather than a morbid symptom to be combated; so I allowed her to take it, and gave her *Stannum* 3 M Jenichen) every four hours.

Oct. 25th. The medicine relieved at first; she had a better night; the suffocation was less, but returned to-day at 11 A. M. The cough was relieved, but increased yesterday, though it is better again to-day. Did not sleep well last night. The flushes of heat were better, but returned since last night. Less sinking and faintness. A little dyspnœa on falling asleep to-day, but much less than formerly. Yesterday afternoon the cutting in the left chest returned, but is now *relieved by lying on the opposite* (*painless*) *side*. With the attacks of loss of breath she has flushes of heat from head to chest, and must draw a long breath. In Lippe's *Repertory* I found "pain in chest worse when lying on affected side" (equivalent to relief when lying on painless side), under *Borax*, *Cact.*, *Calc.*, *Lycop.*, *Sabad.*, and *Sulph.* Of these I selected *Sulph.*, because it (as well as *Laches.*) has a special action on the left upper lung, and gave the DM (F. C.) every four hours.

Oct. 27th. She only took two doses of *Sulphur*, both of which increased the flushes of heat more than ever. After leaving it off she improved, and was better all yesterday. Sleep not good for last two nights. Less cough. No more cutting pain. Dyspnœa unchanged. Just as she is going off to sleep, heat seems to rise up from chest and cause gasping and suffocating feeling. No stool for 11 days, with ineffectual desire. Stronger to-day. Temperature 100.8. Gave no medicine, as she seemed better, and there were no new symptoms of importance.

Oct. 31st. Continued better till last night. The flushes of heat ceased till last night, and then returned only slightly. Very slight cough since last visit. Breathing on the whole better, it still comes on with the heat, as before, on going to sleep. Cutting pain not returned. Bowels acted on 28th, without very much difficulty, and every day since, but only slightly each time. Less dryness of mouth. Did not sleep last night, was light-headed. As the improvement was still continuing, I gave no medicine.

Nov. 2d, 4 P. M. Says the improvement has been ceasing since last night. Hardly any cough, but mucus rattles in chest as she breathes. Bowels relaxed, acting very often, but only a little at each time. No more cutting pain. Not much heat. Dyspnœa a little better; it comes on at any time, not only on falling asleep. Nervous feeling, "as if she could not lie, but could almost fly out of the window," but does not toss about. Mouth dry. Sinking faint feeling in stomach, "as if she were dying right away," since 1 P. M. yesterday, not relieved by food, and preventing sleep. In Lippe's *Repertory*, I found "sinking in stomach after food," and "sleep prevented by emptiness in stomach," under *Dig.* only, so I gave CM (Fincke) every four hours.

Nov. 4th. Took four or five doses at intervals of four hours, and another yesterday afternoon; each dose seemed to increase the sinking feeling. Little cough, but mucus rattles on chest. The night before last was very bad from the sinking. No more cutting pain. Breathing better. Mouth still dry. Costive, stools difficult, scanty. Flushes of heat better. Sinking is better again since leaving off the medicine. As the repetition of the dose had proved too powerful for her, I stopped all medicine.

Nov. 5th, 8 P. M. Sinking feeling is better. No sleep last night from *burning like fire in stomach-pit.* Flushes of heat from head to waist returned. *Thirsty for little and often, craves sour drinks.* Expectoration easier last night. Cutting pains returned once to-day on coughing; cough is only slight. Dyspnœa better last night, but worse this afternoon, so that she had to sit up and breathe short. *The burning makes her feel restless.* Bowels natural to-day. If she lies on either side, she feels a dragging in stomach-pit towards that side, and if she sits leaning back, the stomach-pit feels stretched; she is easiest sitting bent forwards. The italicized symptoms plainly pointed to *Arsenicum,* and I gave her CM (F. C.) every three hours till the burning was better, then less often.

Nov. 6th, 9.30 P. M. The *Arsenicum* relieved her in from half to three-quarters of an hour, and she fell asleep. Had a good night. Burning nearly gone. Flushes of heat less to-day. Thirst unchanged. Much mucus in chest, but little cough. Cutting pain returned twice to-day. Dragging in stomach is better; is now lying on left side. She takes five doses, the last at 2 P. M. She now has intense restlessness, feels as if she must jump up, but cannot rest any way. Breathing is short, quick and panting, *with much action of abdominal muscles;* the expiration is accompanied by a kind of grunt; the dyspnœa prevents her from speaking continuously. Pulse 160. Respiration 70. The breathing has been thus nearly all day. She says she breathes "from the very bottom of the belly." The symptom "abdominal breathing" is unrecorded in any of the published repertories, but in my MS I have recorded it under *Bry.*, *Spong.*, *Tart.*, and *Thuya.* The *Thuya* symptom is found in Wolf's proving; that of *Bry.* and *Tart.* in the *Guiding Symptoms;* the source of the *Spong.* symptom I cannot now discover. As *Spong.* alone of these remedies, had "panting breathing" according to

Lippe's *Repertory*, I gave CM (Fincke). Subsequently on reading *Ant. tart.* in the *Guiding Symptoms*, I concluded that this would have been a more closely indicated remedy; at the time I had prescribed *Spong.* I had not recorded *Ant. tart.* under the heading "abdominal breathing." The *Spongia* seemed to do no good.

Nov. 7th, 10 A. M. No sleep all night from dyspnœa, which has made the muscles of lower abdomen sore and hot. Respiration 72. Pulse 160. Nostrils working somewhat. Intense thirst for little and often, and *it runs audibly down the œsophagus and stomach.* Was nearly choked with the mucus last night. Restlessness worse after 3 A. M. Has had five doses of *Spongia*, the last at 5 A. M. The thirst was worse during last night. The italicized symptom indicated *Cina*, *Cupr.*, *Laur.*, *Thuya* (Lippe's *Repertory*); and as *Thuya* alone corresponded to the nocturnal thirst (Lippe's *Repertory*), and to the abdominal breathing, I gave 15 M (Fincke) every three hours.

Nov. 8th, 1.30 P. M. The medicine quieted the breathing a little; had a quieter night, sleeping better. Is sore from abdomen to throat from the dyspnœa. Thirsty still, and drinks rolls down audibly. Respiration 74. Pulse 168. *Nostrils working more,* and *larynx moving up and down* with the respiration. Abdominal breathing unchanged. The italicized symptoms pointed to *Lycopodium*, of which I gave CM (F. C.) every three hours.

Nov. 10th. Breathing relieved for about half an hour after the first and second doses; then took no more medicine. Very little sleep the last two nights. Respiration 70. Pulse 180. Only a little pain in left upper chest on coughing. Has brought up much thick, white, sticky mucus. Less movement of nostrils. Abdominal breathing a little better. *Pleuritic friction sound in right lower lung, with pain there, which catches her breath.* Flushes of heat gone. Has taken nothing but a little cold tea for three days; it still rolls audibly down throat. *Pressure on left chest still relieves the pain there on coughing.* Face and forehead get cold at intervals. Larynx still works. Feels much weaker. The pleuritic pain, the relief from pressure to the pain of coughing, and the abdominal breathing, indicated *Bryon.*, of which I gave 103 M (F. C.) every six hours.

Nov. 11th. Has had three doses, each of which relieved the pain and dyspnœa temporarily. Now, pulse 150, respiration 60. Abdominal breathing less marked. Slept a little better last night, but with wandering. Coldness of face and forehead still. Repeated *Bryon.* every three hours. She took three more doses, which relieved the pain and dyspnœa, and she died at 11 P. M.

